# Recent Advances in Synthesis, Medical Applications and Challenges for Gold-Coated Iron Oxide: Comprehensive Study

**DOI:** 10.3390/nano11082147

**Published:** 2021-08-23

**Authors:** Mohammed Ali Dheyab, Azlan Abdul Aziz, Mahmood S. Jameel, Pegah Moradi Khaniabadi

**Affiliations:** 1 Nano-Biotechnology Research and Innovation (NanoBRI), Institute for Research in Molecular Medicine (INFORMM), Universiti Sains Malaysia, Pulau Pinang 11800, Malaysia; mahmood@student.usm.my; 2 Nano-Optoelectronics Research and Technology Lab (NORLab), School of Physics, Universiti Sains Malaysia, Pulau Pinang 11800, Malaysia; 3 Department of Radiology and Molecular Imaging, College of Medicine and Health Science, Sultan Qaboos University, P.O. Box 35, Al Khod, Muscat 123, Oman; p.khaniabadi@squ.edu.om

**Keywords:** inorganic nanoparticles, chemical method, formation strategy, medical applications

## Abstract

Combining iron oxide nanoparticles (Fe_3_O_4_ NPs) and gold nanoparticles (Au NPs) in one nanostructure is a promising technique for various applications. Fe_3_O_4_ NPs have special supermagnetic attributes that allow them to be applied in different areas, and Au NPs stand out in biomaterials due to their oxidation resistance, chemical stability, and unique optical properties. Recent studies have generally defined the physicochemical properties of nanostructures without concentrating on a particular formation strategy. This detailed review provides a summary of the latest research on the formation strategy and applications of Fe_3_O_4_@Au. The diverse methods of synthesis of Fe_3_O_4_@Au NPs with different basic organic and inorganic improvements are introduced. The role and applicability of Au coating on the surface of Fe_3_O_4_ NPs schemes were explored. The 40 most relevant publications were identified and reviewed. The versatility of combining Fe_3_O_4_@Au NPs as an option for medical application is proven in catalysis, hyperthermia, biomedical imaging, drug delivery and protein separation.

## 1. Introduction

Coated nanoparticles, or core@shell nanoparticles, consist of two or more nanoparticles that contain a wide variety of organic as well as inorganic nanoparticles, where one serves as a core while the other is centered on the core and named the shell [1]. Knowledge of core@shell synthesis is a pioneering step of nanoscience, as the way to manipulate the nanoparticles’ structure has enabled us to generate a variety of hybrid NPs [2,3]. Core@shell NPs, with the potential to be used as core or shell in a wide variety of materials, will reflect their satisfying distinctive properties and custom functions. Core or shell products can be chosen, depending on the intent of the study [4]. The core@shell property can be modified by causing changes to the components that make up the shell layer or core [5]. Characteristics and distinctive attributes such as optical, magnetic, biological, compatibility, chemical stability and physicochemical properties can be realized when different nanoparticles are incorporated, such as gold nanoparticles (Au NPs) on iron oxide nanoparticles (Fe_3_O_4_ NPs). In recent years, substantial attempts have probably been introduced to evaluate the biomedical applications of Fe_3_O_4_ NPs, including protein purification, immunoassays, hyperthermia, drug delivery, magnetic resonance imaging (MRI), and computed tomography (CT) [6]. Fe_3_O_4_ NPs are the most favored nanomaterials in medical applications because of their minimal toxicity features and excellent physicochemical characteristics such as stability, biocompatibility and supermagnetism [7]. The magnetic response stability of Fe_3_O_4_ is due to its low oxidation sensitivity [8]. In addition, size control, preventing aggregation via coating, precise dispersion and interaction, as well as the penetration of tissue and cell barriers all give Fe_3_O_4_ NPs an advantage over other metal nanoparticles. Fe_3_O_4_ NPs provide a forum for therapeutic uses where they can be utilized for their contrast agent characteristics in MRI diagnostics, as well as for therapeutics in the form of bio-catalysis, drug delivery and protein purification [9].

Various kinds of functional materials, including silica, polymers and Au have been formed on the Fe_3_O_4_ NPs surface to improve biocompatibility, chemical stability as well as processability for broader applications [10,11]. Au is considered to be the most desired coating material for the production of Fe_3_O_4_@Au NPs due to its surface functionality, catalytic activity and superior optical properties [12,13,14,15]. Because of the variety of physicochemical features and the ability to change the magnetic and optical property by modifying the charge, size, shape, surface modification and thickness of the Au shell, Fe_3_O_4_@Au NPs have been widely considered the most effective candidature for medical applications [16]. Several studies have been reported for the synthesis of Fe_3_O_4_@Au NPs. These studies generally described nanoparticles’ physicochemical properties without focusing on a specific formation strategy [17,18,19].

For this reason, the current review will: (1) summarize the latest progress (2018–2020) in the design and synthesis of the Fe_3_O_4_@Au and elaborate upon the strategies involved in the formation Fe_3_O_4_@Au NPs core@shell, Fe_3_O_4_@Au HNPs, Fe_3_O_4_@Au core@satellite NPs as and as nanodumbbells, Fe_3_O_4_@Au DNPs; (2) explore the schemes of each manufacturing strategy for Au-coated Fe_3_O_4_; and (3) present the potency of Fe_3_O_4_@Au as a promising candidature for medical applications in areas of catalysis, hyperthermia, biomedical imaging, drug delivery and protein separation (2018–2020).

## 2. Synthesis of Fe_3_O_4_@Au

Fe_3_O_4_@Au NPs can be classified as Fe_3_O_4_@Au NPs core@shell, Fe_3_O_4_@Au HNPs, Fe_3_O_4_@Au core@satellite NPs as and as nanodumbbells, Fe_3_O_4_@Au DNPs structures. In this section, the synthesis of all structures will be introduced.

### 2.1. Core@Shell Structure of Fe_3_O_4_@Au

**Figure 1:**
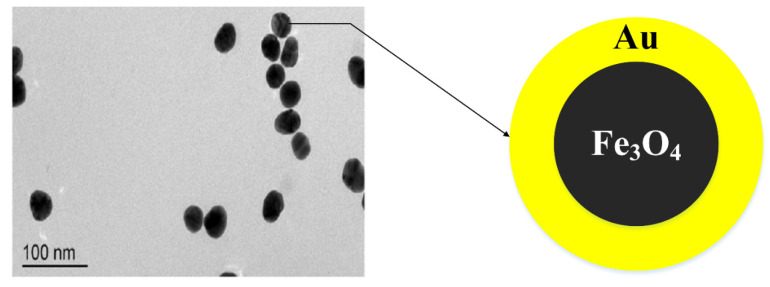
TEM image [20] and schematic diagram of Fe_3_O_4_@Au NPs core@shell construction.

Core@shell nanoparticles have various properties, such as magnetism, metallicity and semiconductivity. These attributes come either through the core or shell materials, or both (Figure 1). In this review, we will discuss Fe_3_O_4_ NPs as a core and Au NP as a shell.

Recently, Xie et al. [21] have synthesized novel core@shell NPs for use in fast, sensitive, convenient and good surface-enhanced Raman scattering. This method involved two steps for the formation of core@shell NPs: (i) the preparation of Fe_3_O_4_@silica NPs, using an ultrasound technique to deposit silica oxide on the surface of Fe_3_O_4_ for 5 min; and (ii) the preparation of the Fe_3_O_4_@SiO_2_@Au seed, using a seed growth method. A similar study was submitted by He et al. [20]. Fe_3_O_4_@Au NPs were coated with glutathione to reduce the dose dependence of the anticancer medication, doxorubicin (DOX), by covering the glutathione shell on Fe_3_O_4_@Au NPs [22]. The former Fe_3_O_4_@Au NPs produce involved this process. Twenty milliliters (20 mL) of HAuCl_4_ solution (0.1%) was added to 40 mL of distilled water in a 250 mL flask. The solution was refluxed and Fe_3_O_4_ was applied to the mixture, then the mixture was boiled for 45 min. A reduction (sodium citrate) was quickly applied under vigorous stirring to the boiling mixture. Citrate addition contributes to the color shift from a grey to a red solution. The solution was boiled for 15 min and then stirred until the solution reached room temperature. The use of glutathione conjugations thus acts as an effective vehicle of drug delivery. In addition to causing drug release with redox-activated glutathione, it requires significantly low levels of glutathione @Au@ Fe_3_O_4_ NPs for DOX releases. The sonochemical approach effectively synthesizes monodispersive and highly stable Fe_3_O_4_@Au NPs, with a size distribution of approximately 20 nm during 8 min [23]. Utilizing surface response (RSM) methodology, test runs of 14 dissimilar variations of gold ions, sonication frequency and sodium citrate (independent variables) have been conducted at two-center points to optimize testing procedures. Variance analysis (ANOVA) has been used to achieve optimal conditions for experimental results. The optimal zeta potential value of about –46.125 mV was reached under the ideal conditions of independent variables, which is compatible (at approximately 99.2%) with the real zeta potential value (–45.8 mV). The monodispersity and stability of the Fe_3_O_4_ NPs effectively coordinated a transition to the core@shell, as demonstrated by a rise in zeta potential value from –24 mV to –45 mV. To date, no work has been reported which produced core@shell NPs for large-scale production. The sonochemical method is widely considered to be one of the most promising methods for preparing, encapsulating and modifying nanoparticles due to its safe, rapid, low-cost and environmentally friendly characteristics [24]. Various types and shapes of nanomaterials have been prepared using a sonochemical method [25,26]. In addition, the sonochemical method ensures the uniformity, homogeneity and monodispersity of the nanoparticles produced [27]. From this point of view, all these advantages and properties of this method may have the potential to be more applicable to large-scale production.

Somayeh et al. [28] carried out a simple and eco-friendly green method for the preparation of spherical Fe_3_O_4_@Au with a size of 31 nm, utilizing the aqueous extract of the Carum carvi seed which plays three functions such as reduction, capping, and stabilizer agents during the Fe_3_O_4_@Au synthesis process (Table 1). The seeds of Carum carvi were thoroughly washed with distilled water, followed by drying at 25 °C for 2 days. In the end, the resultant was milled to produce a powder. In order to prepare the aqueous extract, the powder was steeped in 100 mL of distilled water for 10 h at a temperature of 25 °C and then purified using filter paper to acquire a clear solution. To synthesize Fe_3_O_4_@Au, 50 mg of Fe_3_O_4_ was dissolved in 100 mL of aqueous extract of Carum carvi and the mixture solution was stirred for around 10 min. Twenty milliliters (20 mL) HAuCl_4_ solution (5 mM) was then applied to the mixture solution. Finally, the mixture solution was kept for 24 h and then dried overnight at 70 °C. The green, rapid and low-cost preparation of core@shell Fe_3_O_4_@Au NPs using natural honey as a reducing as well as stabilizing agent through hydrothermal method for 20 min was reported by Rasouli et al. [29]. Fe_3_O_4_ NPs were dissolved in 50 mL ultrapure water and sonicated for 2 min, to which 25 mL of HAuCl_4_ (0.005 M) was added and stirred for 15 min to achieve the full adsorption of gold ions on the surface of Fe_3_O_4_. Subsequently, 0.25 g of the natural honey was added to the mixture solution, held under the hydrothermal method at 120 °C for 20 min. Eventually, Fe_3_O_4_@Au NPs were separated from the excess result solution using a permeant magnet and washed three times through ultrapure water. TEM images revealed that the synthesis of Fe_3_O_4_@Au NPs has a diameter ranging between 3.49 and 4.11 nm. Tarhan et al. [30] announced that novel Fe_3_O_4_@Au NPs, functionalized via maltose, have been prepared as a favorable carrier matrix for easy and efficient L-asparaginase immobilization. The findings show that NPs are monodispersed to 9.0 emu/g magnetization with a size of 10 nm. Tarhan et al. [30] expect that flexible carriers will lead to new possibilities for applications in the fields of biomedicine, biotechnology and biochemistry on the basis of the success of the procedure and the promising findings achieved from their novel process.

Fe_3_O_4_@Au NPs have been produced as novel electrochemical immunosensors for the use of cancer biomarkers [31]. The morphology of Fe_3_O_4_@Au NPs was that of a spherical shape with an average size of approximately 20–50 nm. This novel strategy has shown simpler construction, easier operation and a wider linear range. The proposed approach and the use of a screen-printed carbon electrode provided for the development of a simple electrochemical immunosensor that could be disposable, portable and cheap without using additional labeling. For 15 min under sonication, the suspension of HAuCl_4_ has been stirred with Fe_3_O_4_ solution. Subsequently, the reduction agent solution (NaBH_4_) was quickly added to the cooled suspension, which was then sonicated for another 10 min. Kou et al. [32] reported the custom design of extremely effective catalysts for Fe_3_O_4_@Au NPs. Fe_3_O_4_ was formed with three different morphologies using engineered quantities of urea, and the probable mechanism was proposed. Therefore, by measuring the amount of Au seeds, they achieved Fe_3_O_4_@Au with different morphologies and tunable Au deposition. The catalytical ability of Fe_3_O_4_@Au with several structures was compared through the application to degrade 4-nitrophenol and catalytic rhodamine while systematically investigating the correlation of the Au seed amount to the turnover frequency and the catalytic capability of Fe_3_O_4_@Au. They observed that the flower-like Fe_3_O_4_@Au NPs with 20 mL of Au seeds applied had the highest degradation rate of 96.7%, and after recycling, their catalytic ability was almost unchanged. The formation of Fe_3_O_4_@Au NPs was accomplished by reducing the Au ions on the Fe_3_O_4_ surface using the seeding technique [33]. In a definite volume of glycerin, different concentrations of oxidized Fe_3_O_4_ or the Au-shell reaction were used. The reaction solution, including the reduction agent and Fe_3_O_4_ cores, was first sonicated for 15 min, then heated with vigorous stirring to approximately 150 °C. Once the reaction solution reached 150 °C, a drop-specific solution was added for HAuCl_4_. Fifteen minutes after the addition of Au salts, the heating system was stopped but the stirrer proceeded while the mixture was refreshed at room temperature. The component ratio adaptation allowed the Fe_3_O_4_@Au NPs particle shell thickness to be tuned. The present route produces well-determined structures of the Fe_3_O_4_@Au NPs of various sizes between 15 and 57 nm, with the Au noble metal varying from Fe_3_O_4_ NPs. Bi-phase Fe_3_O_4_@Au NPs were provided using a nano-emulsion technique [34]. Characterization reveals that the Fe_3_O_4_@Au nanostructure produced a particle size and distribution of approximately 11 nm in size. The NPs are non-toxic, water-soluble and stable due to the capping agent covering the particles. Optical and magnetic data indicate that the NPs have a narrow-band surface absorption of plasmon and an increased susceptibility to the Au shell. As a result, the bi-phase Fe_3_O_4_@Au NPs are challenging for various applications such as magnetic separation, optical detection and photonic therapy. In a different process, Au and Fe_3_O_4_ representing magneto-plasmonic NPs were obtained in two successive steps in an aqueous environment by the laser ablation of the Au and Fe_3_O_4_ targets [35]. Au NPs are trapped in a Fe_3_O_4_ mucilaginous matrix, which was established by both microscopic and spectroscopic observation as magnetite. The plasmonic property of the colloids obtained was tested with surface-enhanced Raman scattering spectroscopy, as well as their adsorption capability. In addition to those inherent in Au NPs, the presence of Fe_3_O_4_ offers the bimetallic colloid new avenues of adsorption, particularly with respect to organic contaminants and heavy metals, allowing them to be extracted from the aqueous environment to promote a magnetic field. In addition, these NPs are low in toxicity, making them promising for biomedical applications. Fe_3_O_4_@Au in a size range of about 20–50 nm and significant magnetization saturation using a solvothermal one-pot process was recorded by Ángeles-Pascual et al. [36]. NaBH_4_ gradually reduced the HAuCl_4_ solution into 9 mL of the black NP solution to create a thin gold shell on the Fe_3_O_4_ NPs surface. The solution, under intense stirring, was heated up to 70 °C and allowed to naturally cool down to room temperature. Afterwards, Fe_3_O_4_@Au was separated using the neodymium magnet and rinsed to remove the excess of chemicals from the reagents. To examine the biocompatibility of NPs, a cytotoxicity assay was performed in the MDCK cell line. The tests for the Fe_3_O_4_@Au NPs exhibited higher cell viability, indicating their excellent biocompatibility and their potential for medical application. A novel and direct method for preparing Fe_3_O_4_@Au NPs comprising a Fe_3_O_4_ core coated with an Au shell was identified [37]. The synthesis incorporates ease of operation, minimal control and high reproducibility while at the same time being environmentally friendly. The shell of Au NPs with a controllable thickness of 30 nm was developed on the Fe_3_O_4_ core of 20 nm in size by reducing Au salt in the ultrasonic bath. Au shell thickness might be adjusted by means of varying the quantity of Au salt applied. Fe_3_O_4_@Au NPs of sizes ranging between 80 and 160 nm were prepared. The Fe_3_O_4_@Au NPs were studied for their magnetic and plasmonic behavior. Functionalization with polyethene glycol was conducted to explore its possible use in biomedical applications. Unlike Fe_3_O_4_@Au DNPs, core@shell was commonly utilized as a contrast agent in dual MR and CT imaging techniques.

### 2.2. The Hybrid Structure of Fe_3_O_4_@Au (HNPs)

The synthesis of hybrid Fe_3_O_4_@Au NPs (HNPs) with appropriate size, design and properties is difficult, and has gained considerable attention among researchers in material sciences. It is possible to tune the design of Fe_3_O_4_@Au HNPs by selecting the proper technique and controlling the processing parameters during the synthesis.

Fe_3_O_4_@Au HNPs have single-hybrid nanoparticles consisting of an entire-layer Au ion-reducing coating on the Fe_3_O_4_ surface. In addition to biocompatibility, the structure of Fe_3_O_4_@Au HNPs can also impart the NPs surface with appropriate biological and chemical interface activity [48]. A well-defined novel structure can easily be formed by the Au shell uniformly coated on the surface of Fe_3_O_4_ NPs with sulfur-based ligands. A considerable amount of work has been performed during the last two decades to develop Fe_3_O_4_@Au HNPs using various techniques, including co-precipitation, seed-mediated growth, direct coating and thermal decomposition methods. The most popular method for preparing Fe_3_O_4_@Au HNPs is the Au shell’s direct coating on the Fe_3_O_4_ surface. In this approach, two strategies for forming the shell of Au on the Fe_3_O_4_ surface were observed. The first method is a one-pot process in which the Au ions extend to form the shell on the Fe_3_O_4_ NPs surface. For the second process, Au NPs are internally produced, then seeded into a suspension of Fe_3_O_4_ NPs to create Fe_3_O_4_@Au HNPs [18]. Sood et al. [49] observed that the Au shell’s direct coating on the Fe_3_O_4_ NPs surface loaded with small ligands, including ascorbic acid and citric acid, may be more successful.

**Figure 2:**
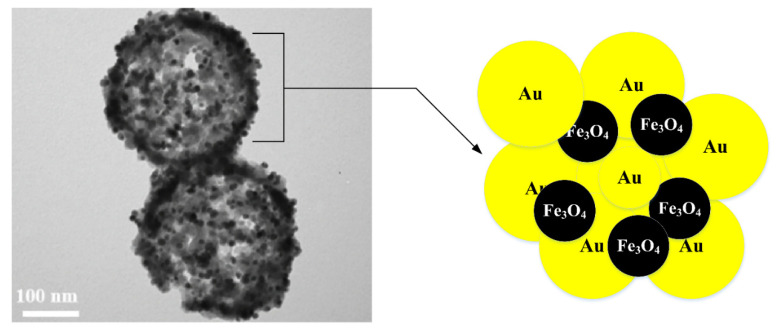
TEM image [39] and schematic illustration for the production of hybrid Fe_3_O_4_@Au HNP.

Park et al. [38] described the hyperthermic features of Fe_3_O_4_@Au HNPs within a 200 kHz and 1.5 kA m^−1^ biocompatible alternating magnetic field (AMF). In the air atmosphere, a 0.4 mL precursor of iron was added to a mixture of both oleic acid with octyl ether at 100 °C. The solution was stirred during 1.5 h before being cooled at room temperature. A mixture of oleylamine (0.5 mmol) and HAuCl_4_ (1.3 mmol) in the 5 mL of chloroform was added two times at intervals of approximately 5 minutes with vigorous stirring with a solvent of oleylamine (2 mmol) and Fe_3_O_4_ NPs (0.1 mg) in chloroform (10 mL). HNPs were produced by growing Au NPs on the Fe_3_O_4_ NPs surface with an average size of 10 nm. Due to the decrease in the saturation value of the HNP solution relative to the Fe_3_O_4_ NPs, the initial heating rate was set to lower than the Fe_3_O_4_ NPs solution. The continued application of the AMF gradually increased the HNP solution temperature, while the solution of the Fe_3_O_4_ NPs achieved thermal equilibrium. A similar AMF condition was demonstrated with the heating efficiency of Au NPs combined with non-conductive and diamagnetic SiO_2_ NPs, which demonstrates that sustained heat for HNPs may be due to the supplementary heating of the Au NPs in a radiation frequency solenoid belt (RF). A novel hollow nanosphere Fe_3_O_4_@Au/polydopamine (Au/PDA) capable of absorbing potentially toxic ions plus catalyzing the decrease in 4-nitrophenol has been published [39]. The hybrid shell has well encapsulated the hollow nanosphere Fe_3_O_4_(Au/PDA) to create the dual-functioning magnetics hollow nanocomposites utilizing an easy redox-oxidizing polymerization technique (Figure 2). Due to its uniform, hollow interior and usable PDA coating with a strong activity of the Au nanoshell, the eventual hollow nanosphere Fe_3_O_4_@Au/PDA has great potential for drug delivery and nanocatalysis. In brief, the multifunctional Fe_3_O_4_@Au/PDA nanosphere has wide application potential for coexisting toxic water contamination, green and simple synthesis and ease of manipulation, effective adsorption efficiency and strong catalytic activity. Au NPs play a crucial part in heterogeneous catalytic reactions. Nevertheless, Au NPs typically have low selectivity and complex recyclability. Fe_3_O_4_@Au@CeO_2_ hybrid nanofibers were prepared in the presence of Fe_3_O_4_ nanofibers, through a simple one-pot redox reaction between HAuCl_4_ and Ce (NO_3_)_3_ [40]. On the Fe_3_O_4_ nanofibers’ surface, the CeO_2_ shell was uniformly coated to form a unique hybrid structure, while the Au NPs were encapsulated within the CeO_2_ shell. As a result of the CeO_2_ shell formation, Fe_3_O_4_@Au@CeO_2_ hybrid nanofibers are positively charged surfaces, allowing them to be excellent choices for the predominantly sensitive catalytic action against the degradation of negatively charged organic colors. The Fe_3_O_4_@Au@CeO_2_ hybrid nanofibers have demonstrated magnetic properties, giving them good recyclable usability. This research provides a simple and efficient solution for preparing the hybrid nanomaterials of magnetic noble metal/metal oxide with a distinctive surface characteristic and chemical structure for offering applications in heterogeneous catalysis. A high temperature wet chemical method was used for the synthesis of Fe_3_O_4_@Au HNPs with a diameter of 25 nm [41]. Fe_3_O_4_@Au HNPs with Au seeds produced in situ were derived at high temperatures through the thermal decomposition of HAuCl_4_ and Fe(CO)_5_. Fe_3_O_4_@Au HNPs revealed the best features for application as hyperthermic and contrast agents for MRI. Due to the large saturation magnetization and octahedral shape of the magnetite particles, Fe_3_O_4_@Au HNPs obtained a particular loss power of approximately 617 W·gFe ^−1^ with an exceptionally high r_2_-relaxivity of about 495 mM^−1^s^−1^.

Wang et al. [42] stated that the novel structure of spiky Fe_3_O_4_@Au (SPs) is used for multi-modal imaging and phototherapy agents. The uniformly sized Fe_3_O_4_@Au SPs were synthesized in two steps. First, citrate-stabilized Fe_3_O_4_ NPs of the average size of 10 nm was synthesized, then the Au layer was coated on the Fe_3_O_4_ NPs surface to create Fe_3_O_4_@Au HNPs, which were used for the production of Fe_3_O_4_@Au SPs. The SPs exhibit great photodynamic effects and therapeutic photothermal, with a photothermal conversion efficiency of about 31%, and enable tumor-targeted imaging, such as MRI, photoacoustic and computed tomography. The SPs display good biocompatibility, in vivo as well as in vitro. Additionally, the SPs obliterated a tumor below 808 nm of radiation owing to its unique absorption in the near-infrared field. SPs represent a convenient product for application in clinical practice with their potential for deeply integrated multi-modal imaging as well as multiple therapeutic functions. Fe_3_O_4_@Au HNPs have been produced, characterized and presented as a new porous marker to increase micro-/nano-based pores found and quantified by SEM in the shale [43]. With the presynthesized Fe_3_O_4_ NPs in a solution, the Fe_3_O_4_@Au HNP shale has been synthesized using the chemical reduction technique. Because of the superparamagnetic properties, the nanomarker is easily operated via the external magnetic field to appoint in pores and provides a sharp contrast picture between the pores and shale matrix, making it much easier and more accurate to recognize micro/nano-sized pores in shales. Moreover, as Au NPs are particularly rare noble metals in the shale, Au’s energy-dispersive X-ray mapping was used to accurately calculate area porosity in a shale. A precise and realistic technology is recommended to enable the characterization of micro/nano-pores in the shale in conjunction with the aforementioned merits of the nanomarker. The design and synthesis of hybrid NPs with distinct morphologies can draw the interest of scientists to hybrid biosynthesis NPs.

### 2.3. Core@Satellite Structures

**Figure 3:**
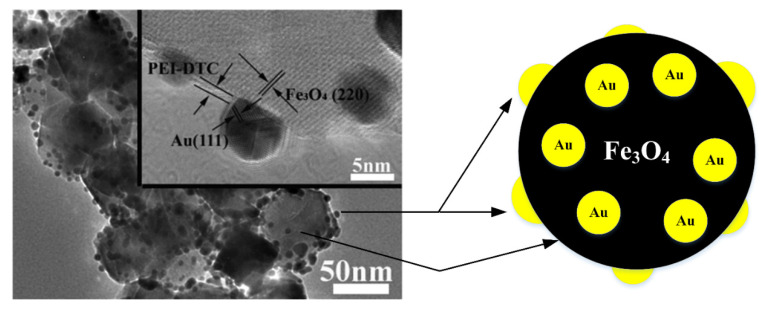
TEM image [44] and schematic diagram for the preparation of core@satellite CsFe_3_O_4_@Au NPs.

One of the popular frameworks for Fe_3_O_4_@Au NPs is core@satellite (Cs). This structure has a single core of Fe_3_O_4_ with the binding by covalent bonds of numerous Au NPs similar to satellites. The CsFe_3_O_4_@Au NPs comprise a residually exposed core surface of Fe_3_O_4_ suitable for MR imaging and further functionalization. In addition, the Cs structure consists of many peripheral Au NPs with a large surface area of the satellite nanoparticle that is advantageous for imaging as well as photothermal capabilities [50]. CsFe_3_O_4_@Au NPs are drawn up using different methods. Liu et al. [44] announced that a seed deposition method was used to produce CsFe_3_O_4_@Au nanocubes (Figure 3). Ten milliliters (10 mL) of Au seeds were applied dropwise to obtain Fe_3_O_4_@PEI nanocubes dispersed in deionized water through ultrasonic treatment. The CsFe_3_O_4_@PEI@Au nanotubes were thoroughly washed with deionized water after 2 h of sonication. Recently, Song et al. [45] succeeded in developing CsFe_3_O_4_@Au NPs that combined three-dimensional microporous graphene foam was formed by an efficient approach which integrated in situ growth, hydrothermal treatment and freeze-drying methods. Ultrasonic treatment was required during the sample preparation to help form a stable mixed colloidal suspension of precursors. Nevertheless, it is notable for Au NPs to be removed from the CsFe_3_O_4_/Au NPs by using ultrasound. The binding force between the products of CsFe_3_O_4_/Au NPs must be powerful enough to solve this problem. As a result, the Cs Fe_3_O_4_/Au NPs used in this method were provided using an in situ growth technique, where Fe_3_O_4_ NPs coated with citric acid were utilized as seeds to reduce gold ions (HAuCl_4_) with the asset of sodium citrate for the nucleation and growth of Au NPs on Fe_3_O_4_ NPs surfaces.

### 2.4. Fe_3_O_4_@Au Nanodumbbells

**Figure 4:**
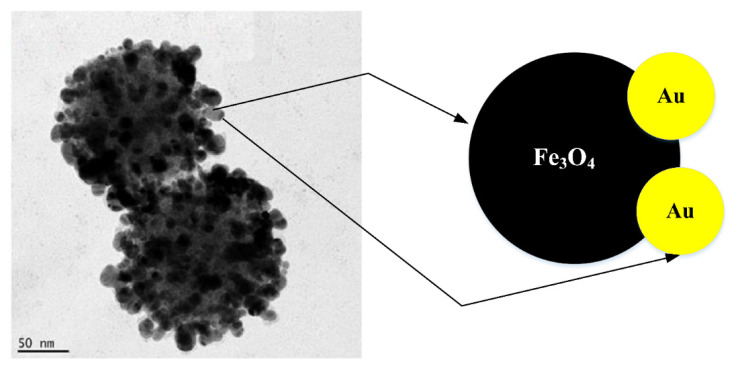
TEM image [55] and schematic drawing of Fe_3_O_4_@Au DNPs dumbbell preparation.

Dumbbell NPs (DNPs) consist of a tightly interacting heterostructure together with one NP at the other end (Figure 4). The separate NPs are dumbbell-like or resemble particles in near contact with each other. In contrast to Fe_3_O_4_@Au HNPs in which Au shields the Fe_3_O_4_ core, the Fe_3_O_4_@Au DNP’s have a broad-based functional surface and active interface which improves their applications for diagnostics and therapy as theranostics [51]. Fe_3_O_4_@Au DNPs have unique features, including (1) the ability to allocate various functionalities to delivery applications and particular target imaging; (2) the magnetic detection and simultaneous optical abilities; and (3) the ability to customize optical and magnetic features by adjusting the size of Fe_3_O_4_@Au HNPs [52]. Fe_3_O_4_@Au DNPs can be regularly produced through the epitaxial growth of one NP to another form of NPs called NP seed. During the procedure, the nucleation should be properly regulated to generate heterogeneous nucleation on a particular crystalline phase around the seed NPs [53]. Klein et al. [46] developed a simple one-pot synthesis method for the preparation of Fe_3_O_4_@Au DNPs using a sonication process. In their analysis, Fe_3_O_4_@Au DNPs were achieved by the co-precipitation of Fe_3_O_4_ NPs in an aqueous solution of HAuCl_4_. Subsequently, 3-mercaptopropionic acid was added to a mixture to stabilize Fe_3_O_4_@Au DNPs. The resulting DNPs were collected by permanent magnetism and washed three times with 20 mL of ultrapure water. Kostevsek et al. developed Fe_3_O_4_@Au DNPs coated with chitosan using a two-step process [47]. First, Fe_3_O_4_@Au DNPs were provided through the reduction of Au ions using the thermal decomposition of the Fe pentacarbonyl (Fe(CO)_5_) with the existence of oleic acid, oleylamine and 1,2-hexadecanediol at the same time. An Au NP was observed to develop at first in the mixture, during the reaction because of a larger variance in the potential for reduction between Fe and Au. Afterwards, Au NPs were used to break down Fe(CO)_5_ to produce Fe_3_O_4_@Au at higher temperatures. Second, the surface of Fe_3_O_4_@au presynthesized NPs was changed to produce highly biocompatible Fe_3_O_4_@Au DNPs coated with chitosan, utilizing hydrocaffeic acid and thioglycolic acid-conjugated chitosan. Fe_3_O_4_@Au DNPs were shown to be biocompatible within a certain range of concentrations that can be employed for optical and magnetic applications in biomedicine [54]. Despite the fact that much work has been expended in the synthesis of Fe_3_O_4_@Au CNPs for MR@CT imaging applications, the synthesis and development of these nanoparticle systems remain an open area with significant challenges. For example, Fe_3_O_4_@Au nanodumbbells have not been extensively used for dual-mode MR/CT imaging applications.

## 3. Medical Application of Fe_3_O_4_@Au NPs

**Figure 5:**
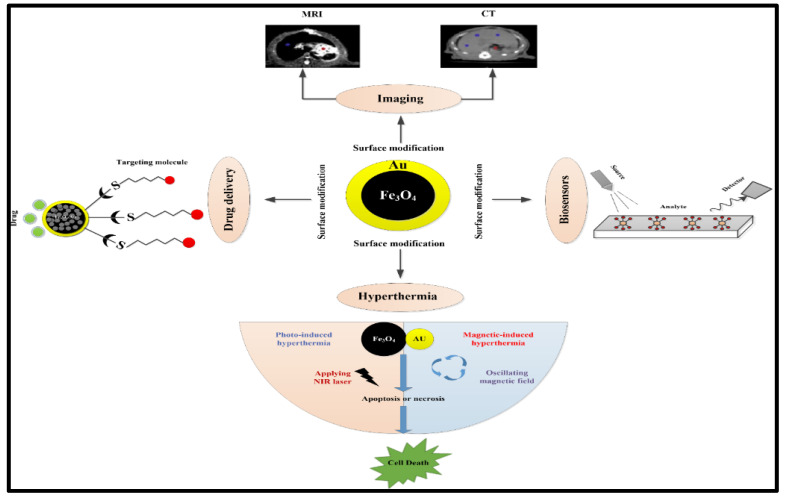
Schematic representation of the medical applications of Fe_3_O_4_@Au NPs [5].

Nanoscience currently ranks among the world’s most desirable sciences due to its interdisciplinary research field, which can be used in many applications [55,56]. Fe_3_O_4_@Au with enhanced properties possesses a specific economic value relative to single NPs due to the current increase in performance, durability and a wide range of industrial, engineering and medical applications. Recently, Fe_3_O_4_@Au NPs have attracted many researchers due to their wide variety of features, potential, structures, easy control and simple production methods, as discussed above. Fe_3_O_4_@Au NPs were employed for a wide range of applications, including catalysis [57], hyperthermia [58], biomedical imaging [59], drug delivery [29] and protein separation [60]. Thereby, the most desirable applications will be discussed (Figure 5).

Izadiyan et al. [61] documented the construction of Fe_3_O_4_@Au NPs using a modern two-step synthesis technique made of green husk extract from Juglans regia. Their analysis shows that Fe_3_O_4_/Au NPs’ structure, physical and chemical properties exhibit Fe_3_O_4_ and Au’s intrinsic features. The Fe_3_O_4_@Au NPs display 235 μg/mL of inhibitory concentration (IC)_50_ against colorectal cancer cells (HT-29). Once measured against non-cancer cells, not even up to 500 μg/mL of IC_50_ was obtained. This result exhibited the promising properties of Fe_3_O_4_@Au NPs for cancer treatment and different biomedical applications. Au shell coating over Fe_3_O_4_ NPs provides an appropriate platform for adequate modification via therapeutic agents, which is one of the main challenges for the use of Fe_3_O_4_@Au NPs through cancer therapy. Cancer cells lack the necessary heat-shock reaction and therefore start dying before normal cells when the temperature of the tissue is above 42 °C, and the time necessary to achieve the therapeutic temperature was indeed faster for Fe_3_O_4_@Au NPs than for naked Fe_3_O_4_ NPs [36].

**Figure 6:**
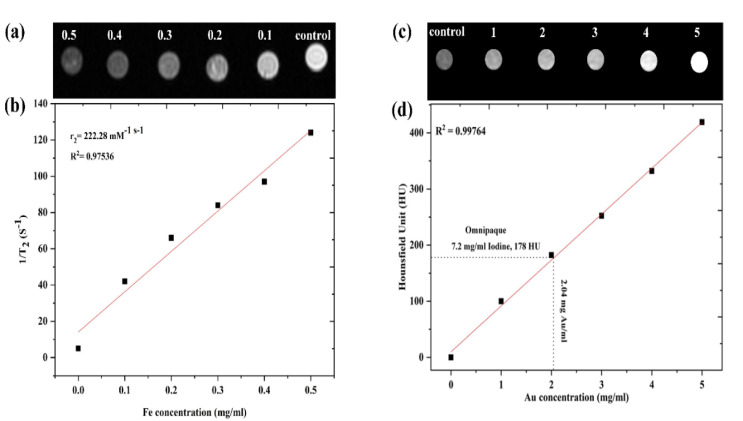
(**a**) MR images with various Fe doses; (**b**) T_2_ linear fitting of Fe_3_O_4_@Au NPs; (**c**) CT images of various Au doses; and (**d**) the intensity of X-ray attenuation [63].

Zhao et al. [62] reported the production of Fe_3_O_4_@Au HNPs at room temperature, which concurrently improved X-ray attenuation as well as showed fluorescence and magnetic properties. Findings from the in vitro fluorescence experiment revealed that the NPs were extremely photostatic and could prevent endosome degradation in cells. Additionally, the in vivo study of normal mice showed 34.61 times more contrast under MR guidance 15 min after the administration of the Fe_3_O_4_@Au HNPs. The most elevated Hounsfield unit (HU) stood at 174 for 30 min after injections of Fe_3_O_4_@Au HNPs by CT. In vivo studies of Fe_3_O_4_@Au HNPs in rat models carrying three different viral infections were further evaluated. For fatty liver models, almost constant contrast improvement was observed without focus dysfunction or nodules under CT and MR (72 HU) and (the highest contrast ratio was 47.33). At the same time, the pronounced enhancement of HCC and cirrhotic liver under CT and MR guidance might be observed in liver parenchyma following Fe_3_O_4_@Au HNPs injection with highlighted lesions. In addition, the biochemical, hematological and pathological analysis revealed a lack of chronic and acute toxicity and demonstrated the biocompatibility of Fe_3_O_4_@Au HNPs in vivo applications. These Fe_3_O_4_@Au HNPs have shown great potential as a bio-image and multi-modality candidate. Recently, our team recorded Fe_3_O_4_@Au NPs developed through sonochemical production for MR and CT imaging [63]. The Fe_3_O_4_ NPs were produced by co-precipitation, followed by the reduction of a gold ion on the Fe_3_O_4_ surface utilizing a simple and rapid sonochemical process, in just 10 min. Viability testing for a human embryonic kidney cell line (HEK-293) with various doses (100 to 500 Fe μg/mL) for Fe_3_O_4_ and also Fe_3_O_4_@Au was performed for various incubation periods (24, 48 and 72 h). Significant reduction in the viability of HEK-293 cells could indeed be identified through an increase in the NPs dose. HEK-293 cells were cultivated with various concentrations of coating NPs (Fe_3_O_4_@Au), which were higher than that of bare Fe_3_O_4_ due to the biocompatibility properties of the Au shell. This result means that the Au shells could decrease the toxicity of Fe_3_O_4_ [64]. Fe_3_O_4_@Au NPs were first distributed as a control sample in various agar gel concentrations (0.1 to 0.5 mg) using a simple agar gel (Figure 6a). The brightness of the Fe_3_O_4_@Au NPs MRI images reduces if the dose increases, leading to a decrease in the MRI signal strength via the increasing Fe dose [65]. Transverse relaxivity (r_2_) is typically used as a contrast agent to measure the effectiveness of Fe_3_O_4_. Illustration 6 (b) provides a relaxation rate (T_2_) as a variable of the Fe_3_O_4_@Au NP dose in which T_2_ linearly increases during the increase in the Fe dose with an r_2_ slope value of about 222.28 mM^−1^ s^−1^ (Table 2). Fe_3_O_4_@Au NPs’ r_2_ value is high, probably due to the water protons that can be obtained at the Fe_3_O_4_ surface of the shell during the interstitial spaces of Au shells. The result of the sensitivity supports the possible use of Fe_3_O_4_@Au NPs in MRI applications as a T_2_-shortening agent. The X-ray attenuation of various concentrations of Fe_3_O_4_@Au NPs has been studied, employing agar as a sample group to assess the potential of the use of Fe_3_O_4_@Au NPs as a contrast agent for CT (Figure 6c). The sensitivity of the CT picture improves with the concentration of Au. Illustration 6 (d) exhibits that the Fe_3_O_4_@Au NPs’ CT value (HU) gradually increases with the concentration of Au shell (HU = 418) [66]. This report reveals that the attenuation rate of Fe_3_O_4_@Au under parallel concentrations of iodine is significantly higher than Omnipaque. This reduction was consistent with an earlier report [66]. Sun et al. stated that because of their higher surface-to-volume ratio, ultrafine Au shells demonstrate higher X-ray attenuation compared to their larger equivalents [67]. This function is imperative since Fe_3_O_4_@Au NPs’ strong X-ray attenuation capability is a prerequisite for their future utilization as a CT contrast agent. In vitro results (r_2_ and HU) support the efficacy of Fe_3_O_4_@Au in MR and CT imaging. In general, Fe_3_O_4_@Au NPs’ MRI contrast influence depends on Fe_3_O_4_ concentration, whereas the Au shell serves an essential function via the X-ray attenuation of CT imaging.

Mohammed et al. [68] announced that the sonochemical method successfully synthesized Fe_3_O_4_@Au with a mean size of 20.8 nm. Fe_3_O_4_@Au NPs demonstrated slight toxicity to MCF-7 cell lines within 24 h, even with the maximum NPs concentration. The laser irradiation time, power, and wavelength used to treat both cells and NPs were 10 min, 200 mW and 808 nm, respectively. Cell viability decreased dramatically after treatment with 50 µg Fe/mL Fe_3_O_4_@Au NPs. The findings in this study conclude that Fe_3_O_4_@Au NPs have the ability to be used as a phototherapeutic agent to improve breast cancer treatment. Fe_3_O_4_@Au NPs were designed for a plasmon signal enhancement label for nucleotide and serum marker combined detection by Premaratne et al. [69]. The Fe_3_O_4_@Au NPs’ integrated plasmon and magnetic enhancement features proved capable of quickly and magnetically separating the detection-attached sensors and magnifying the SPR signal’s performance whilst reducing the non-particular signals of a serum matrix. Such features enhanced the assay’s dynamics as well as its selectivity and sensitivity. With the recently developed emphasis on in vitro diagnostic imaging for painless/non-invasive disease and abnormality detection, results showed Fe_3_O_4_@Au NPs to be new multiplex biosensors of real laboratory testing in complex matrices. Spiky Fe_3_O_4_@Au NPs were proven to be efficacious theranostic agents in photothermal treatment, a drug-targeted delivery and genetic transmission system [70]. The clearance, biocompatibility and biodistribution of the spiky Fe_3_O_4_@Au were studied in mice. The organ distributions revealed that the intravenously administered spiky Fe_3_O_4_@Au NPs were mainly accumulated in the spleen and liver, and the size of the particles significantly affected their actions in vivo. The biochemistry and electron transmission microscopy serum of ultra-histologic structures revealed that spiky Fe_3_O_4_@Au NPs had no significant in vivo toxicity and did not present a potential risk of kidney and liver dysfunction. Such results lay the groundwork for the development of future theranostic agents. Kang et al. [71] studied the dual-mode imaging of Fe_3_O_4_@Au NPs as contrast agents for magnetic resonance (MR) and photoacoustic (PA) imaging. MR imaging offers a time-dependent location for the tumor, while PA imaging demonstrates the presence of high-resolution blood vessels within the tumor. The Fe_3_O_4_@Au NPs display a greater value of r_2_—approximately 329 mM^−1^ s^−1^. The Fe_3_O_4_@Au NPs were also added to the tumor-bearing mice of LNCaP as a successful candidate to image tumors for Vivo PA/MR through intravenous injection. MR/PA imagery results in the tumor area show a substantially improved MR/PA image. In multi-modal imaging, the prepared Fe_3_O_4_/Au NPs will be widely applied. Fe_3_O_4_@Au NPs were synthesized through the chemical reduction approach [72]. TEM analysis revealed the production of Fe_3_O_4_@Au with a mean size of approximately 18 nm. The decreased size allows these Fe_3_O_4_@Au NPs to effectively infiltrate the bacterial crust, resulting in membrane reliability failure. These Fe_3_O_4_@Au NPs indicate high antibacterial activity in the water against Gram-positive and Gram-negative pathogens. The result achieved showed that the Fe_3_O_4_@Au NPs were a strong antibacterial agent. Using invented Fe_3_O_4_@Au NPs in the medical industry is still challenging because the results of clinical trials have yet to be released.

## 4. Conclusions and Challenges

Fe_3_O_4_@Au NPs provide numerous possibilities for a powerful platform for medical applications due to their special optical and magnetic properties. Owing to advances in synthesis methods, various forms of Fe_3_O_4_@Au NPs such as core@shell NPs, core@shell HNPs, core@satellite NPs and dumbbell NPs have recently been explored. The physicochemical characteristics of Fe_3_O_4_@Au were controlled by manipulating each NP in terms of composition, size, shape and interparticle correlations according to their needs. Fe_3_O_4_@Au NPs have been commonly regarded as therapeutic agents for various uses due to various functional materials, including catalysis, hyperthermia, biomedical imaging, drug delivery, and protein separation. Nonetheless, the use of Fe_3_O_4_@Au NPs as a medical agent is still in its infancy and is faced with many doubts and challenges. It is very challenging to develop more effective, smart and secure Fe_3_O_4_@Au NPs for medical applications. While several Fe_3_O_4_@Au NPs have been established, translating these components into real clinical applications has not yet been carried out. To address these drawbacks, efforts should be made to produce Fe_3_O_4_@Au NPs, where each functionality performs in a combined way without affecting other features and functionality. In addition, these components should be precisely applied to long-term toxicity investigations, biodistribution evaluation and several other preclinical tests. Despite these challenges, medical applications based on Fe_3_O_4_@Au NPs will indeed find real-time applications due to their special features. Collective efforts from researchers from multidisciplinary backgrounds can enhance the success of using Fe_3_O_4_@Au HNPs as a medical agent.

## Figures and Tables

**Table 1:** Summary of the recently published studies on the synthesis methods of Fe_3_O_4_@Au NPs.

No.	Nanoparticles Structure	Synthesis Method	Size/Shape	Applications	Ref
1	Core@shell	Growth method	5 nm/spherical	Food application	[21]
2	Core@shell	Sonochemical	~40 nm/flower	Food application	[20]
3	Core@shell	Green method	31/spherical	Antimicrobial activity	[28]
4	Core@shell	Green method	3.49–4.11 nm/semispherical	Drug delivery	[29]
5	Core@shell	Reduction	10 nm/amorphous	Enzyme immobilization	[30]
6	Core@shell	Sonochemical	20–50 nm/spherical	Cancer biomarkers	[31]
7	Core@shell	Seeds growth	9.49, 10.04 and 8.95 nm/flower	Catalytic reduction of RhB	[32]
8	Core@shell	Seeding technique	15–57 nm/		[33]
9	Core@shell	Nano-emulsion technique	11 nm/semispherical		[34]
10	Core@shell	Laser ablation	20 nm/spherical		[35]
11	Core@shell	Reduction	20–50 nm/semispherical	Cytotoxicity assay in MDCK cell line	[36]
12	Core@shell	Reduction	~100 nm/flower		[37]
13	HNPs	Reduction	10 nm/spherical	Hyperthermia	[38]
14	Au/PDA hybrid	In situ redox-oxidize polymerization	25 nm/spherical	Catalysis and adsorption	[39]
15	Fe_3_O_4_@Au@CeO_2_ hybrid	Redox reaction	17 nm/nanofibers	Catalysis	[40]
16	HNPs	Thermal decomposition	25 nm/octahedral	Theranostics	[41]
17	HNPs	Seeds growth	90 nm/spiky	Multimodal in vivo imaging	[42]
18	HNPs	Chemical reduction	31 nm/spherical		[43]
19	Core@satellite	Seed-mediated growth	65 nm/cubic	Catalysis	[44]
20	Core@satellite	Hydrothermal treatment, and freeze-drying technologies	300–400 nm/spherical	Microbial fuel cells	[45]
21	Dumbbell NPs	Reduction	22 nm/spherical	Radiation therapy	[46]
22	Dumbbell NPs	Thermal decomposition	7 nm/spherical		[47]

**Table 2:** Summary of the recently published studies on medical applications of Fe_3_O_4_@Au NPs.

No.	Nanoparticles Type	Application	Results	Ref
1	Core–shell Fe_3_O_4_/Au	Anticancer	The Fe_3_O_4_@Au NPs display 235 μg/mL of inhibitory concentration (IC)_50_ against colorectal cancer cells (HT-29).	[61]
2	Fe_3_O_4_@Au HNPs	CT-MR dual-modality contrast agents	In vitro phantom studies revealed that these NPs provided superior contrast enhancement for CT and MR imaging.	[62]
3	Fe_3_O_4_@Au NPs	MRI and CT imaging	The in vitro findings (r_2_ = 222.28 mM^−1^ s^−1^, HU = 418) substantiate the effectiveness of Fe_3_O_4_@Au NPs in MRI and CT imaging.	[63]
4	Fe_3_O_4_@Au NPs	Photothermal therapy	The findings demonstrated that Fe_3_O_4_@Au NPs have the ability to be used as a phototherapeutic agent to enhance the eradication of breast cancer cells.	[68]
5	Fe_3_O_4_@Au core/shell	Biosensors	Fe_3_O_4_@Au NPs as new multiplex biosensors of real laboratory testing in complex matrices.	[69]
6	Spiky Fe_3_O_4_@Au NPs	Theranosticagents	The serum biochemistry results showed that the spiky Fe_3_O_4_@Au NPs had no discernible toxicity in vivo and could not accurately depict liver and kidney failure.	[70]
7	Fe_3_O_4_@Au NPs	Dual-modal imaging	The Fe_3_O_4_@Au NPs proved to be a successful candidate to image tumors for Vivo PA/MR through intravenous injection.	[71]
8	Fe_3_O_4_@Au NPs	Antibacterial study	Fe_3_O_4_@Au NPs revealed good antibacterial activity against Gram-positive and Gram-negative pathogens which are found in water.	[72]
